# Predictive factors of cervical nodal metastases in N0 squamous cell carcinomas of the mobile tongue – A cohort study

**DOI:** 10.1016/j.amsu.2020.10.068

**Published:** 2020-11-10

**Authors:** Yassir Hammouda, Khadija El Bouhmadi, Omar Iziki, Youssef Oukessou, Sami Rouadi, Redallah Larbi Abada, Mohamed Roubal, Mohamed Mahtar

**Affiliations:** Department of Otorhinolaryngology, Head and Neck Surgery, Ibn Rochd Hospital, King Hassan II University, Morocco

**Keywords:** Carcinoma, Metastases, Tongue, Predictive factors

## Abstract

Squamous cell carcinoma (SCC) of the tongue is one of the most common cancers in the oral region, most frequently associated with lymph nodes metastases which influence the most the prognosis. The identification of predictive factors of occult cervical nodal metastases for N0 tumors will allow to adapt the treatment to the patient, avoiding over or under management.

From 2014 to 2019, a cohort of 26 patients with SCC of the mobile tongue was reviewed by analysing the medical history, the epidemiological and clinical parameters, the tumor sites, aspects, diameters, depths of invasion, pathological degree, degree of differentiation, T classification and results of neck dissections.

The incidence of occult cervical nodal metastases was up to 26,92% and a significant correlation was only found with the tumor depth invasion and the muscular invasion (p < 0,05).

Presently, a low differentiated, highly graded tumor with a high depth and muscular invasion should warn from the high incidence of occult cervical nodal metastases and should recommend an elective neck dissection in all cases of N0 tongue SCC.

## Highlights

• The tongue is the most deadly location of oral SCC and most commonly associated with nodal metastases.
• The depth of invasion and muscular infiltration seem to influence the occurrence of nodal metastases.
• Elective dissection (level I, II, III) with tumour excision is the effective treatment of N0 SSC of the tongue.

## 1 Introduction

The cancer of the tongue represents 47% of all oral cavity cancers in our practice according to the Casablanca local cancer register. With the squamous cell carcinoma (SCC) or epidermoid carcinoma as the most common type, up to 90%. The nodal status remains the most important prognosis factor. But, if the management of N+ tumors is agreed and consensual based on the neck dissection, for the N0 stages is still controversial between the surgical dissection or the medical supervision alone, or even, as the most recent option, the histological analysis of the sentinel lymph node.

Our study aims to evaluate the nodal status of the SCC of the mobile tongue classified as N0 as well as the benefit of the neck dissection in this indication, in one hand; and, in another hand, to identify the predictive factors of occult nodal metastases on these patients to avoid overtreatment or undertreatment.

### 1.1 Patients and methods

A retrospective review was carried out based on the analysis of the medical records of a cohort of 26 patients treated at the Department of Head and Neck Surgery of the Ibn Rochd hospital of Casablanca, between January 2014 and January 2019, for a SCC of the mobile tongue with no clinical or radiological lymphadenopathies (cN0). The clinical and pathological data of all the patients were complete. All the patients underwent a total resection of the tumor with an elective neck dissection of the levels I, II and III. No particular pre-intervention considerations were taken and the post-intervention follow up went well.

The medical history and epidemiological and clinical parameters were stated. The tumor sites, aspects, diameters, depths of invasion, pathological degree, degree of differentiation, T classification according to the 2017 revised 8th edition of the TNM classification and results of neck dissections were reviewed. The correlation between occult nodal metastases and these factors was analyzed.

The statistical analysis leading to the correlations found between the clinical et pathological factors and the occurrence of occult cervical nodal metastases was based on the khi-2 test of Pearson, considering the correlation significant with p < 0,05.

The work has been approved by the ethical committee of our department. All the patients gave their consent for the surgery and the follow up leading to the results of this study which is registered under the number 5887 on the Research Registry. The paper was written meeting the STROCSS criteria [1].

## 2 Results

• **Clinical features**

Amongst the 26 patients collected, there were 16 males and 10 females, with a sex ratio up to 1,6. The median age was 56 years (ranged from 37 to 83 years). The consultation time varied from 1 month to 3 years after the discovery of the lesion, with a median at 8 months.

The risk factors noted were as follow: smoking (42%), alcohol drinking (12%), poor dental condition (30%) and dental prosthesis wearing (10%).

The clinical examination found no lymphadenopathies, as well as the radiological examination which consisted on a cervical ultrasound for 11,5% of the patients, a cervicofacial computed tomography for 53,8% and a cervicofacial MRI for 57,7%.

• **Tumor characteristics**

The clinical aspect of the tumor was a budding form in 24% of cases, an ulcerative form in 22%, a budding and ulcerative form in 46% and an infiltrative form in 8% of the cases.

**Fig. 1:**
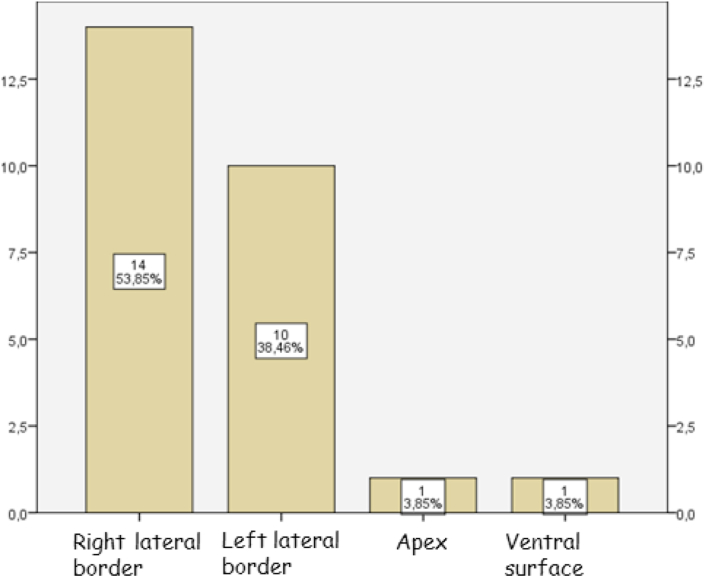
Distribution of the patients according to the tumor sites. Of the 26 patients, 5 had a T class of T1, 17 a T class of T2, 3 a T class of T3 and 1 a T class of T4 (Fig. 2).

The tumor sites included the right lateral border for 14 patients, the left lateral border for 10 patients, the apex of the tongue for one and the ventral surface for another one (Fig. 1).

• **Nodal yield:**

**Fig. 2:**
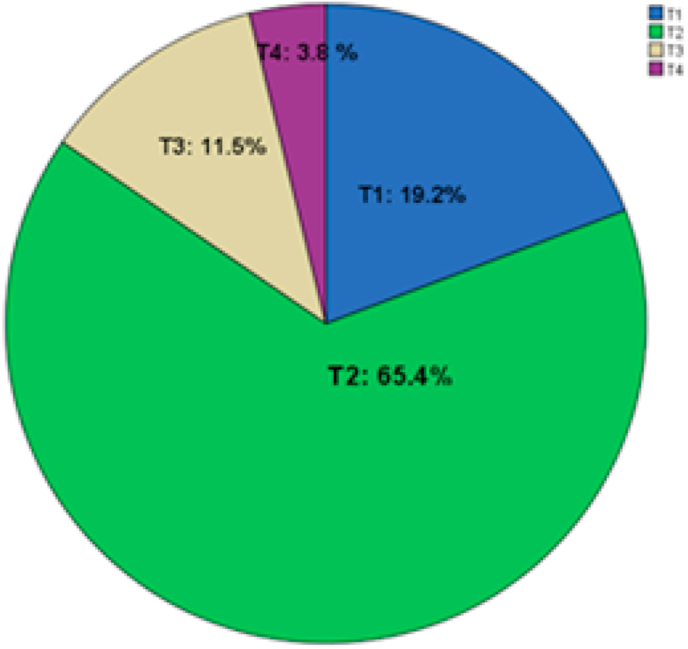
Distribution of the patients according to the T class.

For all the 26 patients, was performed a resection of the primary tumor with an elective neck dissection interesting the level I, II and III, on the right neck in 53,8% of the cases, on the left in 34,6% and on the both sides in 11,5%. No surgical complication has occurred.

In total, 277 nodes were examined histologically, with a mean number of 10,6 per dissection, and 21 were positive for metastatic carcinoma. These pN+ were found on 7 patients. Thus, the incidence of occult cervical nodal metastases on our clinical and radiological N0 patients was up to 26,92%.

• **Correlation between occult cervical nodal metastases and clinical factors:**

The following table shows the correlation between each clinical parameter and the occurrence of nodal metastases (Table 1).

**Table 1:** The correlation between each clinical parameter and the occurrence of nodal metastases.

Clinical features	Number of cases	Occult nodal metastases	Percentage	p
**Gender**				
Male	16	4	25%	0,235
Female	10	3	30%	
**Age**				
<50 years	7	2	29%	0,235
>50 years	19	5	26%	
**Smoking**				
Yes	11	2	18%	0,39
No	15	5	33%	
**Time of consultation**				
<8 months	16	4	25%	0,62
>8 months	10	3	30%	
**Site of the tumor**				
Right lateral border	14	3	21%	0,117
Left lateral border	10	2	20%	
Apex	1	1	100%	

Ventral surface	1	1	100%	
Dorsal surface	0	0	0%	
**Aspect of the tumor**				
Budding	6	1	17%	0,089
Ulcerative	7	2	28%	
Ulcerative and budding	11	2	18%	
Infiltrative	2	2	100%	
**T Class**				
Tis	0	0	0%	0,621
T1	6	1	17%	
T2	15	4	26%	
T3	4	2	50%	
T4	1	0	0%

**Table 2:** The correlation between each pathological parameter and the occurrence of nodal metastases.

Pathological features	Number of cases	Occult nodal metastases	Percentage	p
**Depth of invasion** <0,7 cm 0,8–1,5 cm >1,5 cm	12 8 5	0 3 4	0% 37,5% 80%	**0,03**
**Degree of differentiation** Well differentiated Moderately differentiated Poorly differentiated	24 2 0	6 1 0	25% 50% 0%	0,444
**Muscular infiltration** Yes No Unspecified	11 14 1	7 0 0	64% 0%	**0,01**
**Vascular emboli** Yes No Unspecified	8 14 4	1 3 3	12,5% 21%	0,056
**Perineural invasion**	–	–	–	–
**Mitotic index**	–	–	–	–

It appears that there was no significant difference in the occurrence of nodal metastases in relation to sex (p = 0,23), age (p = 0,23), smoking (p = 0,39), time of consultation (p = 0,62), site of the tumor (p = 0,11), its aspect (p = 0,08) or its T class (p = 0,62).

• **Correlation between occult cervical nodal metastases and pathological factors:**

The following table shows the correlation between each pathological parameter and the occurrence of nodal metastases.

There was a significant positive correlation between the tumor depth invasion and the percentage of appearance of nodal metastases since no N+ was noticed when the tumor did not exceed 0,7 cm in depth. While the rate of metastases increased to 37,5% for tumors between 0,8 and 1,5 cm, and reached 80% for the tumors over 1,5 cm.

Also, the muscular invasion seemed to be an influencing factor since its presence raised the rate of occult nodal metastases from 0 to 64%.

On another hand, no significant difference was noticed concerning the degree of differentiation of the tumor or with the presence or absence of vascular emboli.

## 3 Discussion

The tongue is considered as an aggressive location of SCC given that the tongue possesses a rich vascular and lymphatic network and a well-represented musculature, which would explain the tendency to invasion and regional metastasis [2]. Besides, tongue SCC appears to be most commonly associated with lymph nodes metastases and thus, the most deadly of the topographic locations of oral SCC [3,4], while the rate of occult metastases can reach 30–34% according to literature for tumors of the oral cavity and oropharynx [5]. In fact, the presence of these metastases is one of the most important prognostic factors for tongue SCC survival and the presence of extracapsular spread worsens the prognosis [3,6].

Presently, there are three therapeutic options for the N0 SCC of the tongue. Either a functional neck dissection or elective radiotherapy, or a superomohyoid neck dissection that can be switched to a radical neck dissection if the examined doubtful metastases turn positive, or, at last, the “wait and see” option based on surveillance, reserving surgery for when metastatic lymph nodes are observed, avoiding overtreatment [7]. But, controversy still exists over which option to follow and various authors studied the question coming to different conclusions.

Concerning the therapeutic protocol of patients with early stages of SCC of the tongue (cT1 T2N0), a meta-analysis by Abu-Ghanem S et al. reviewed 23 studies from 13 countries showed that elective neck dissection significantly reduced the risk of nodal recurrence in comparison with a management by observation. Also, after analysing four studies related to the disease specific survival rate, a 51% benefit was observed with the elective neck dissection [8].

While the incidence of occult metastases on our study was up to 26,92% on a total of 26 patients, Ebrahim et al. found an incidence of 37% over 153 patients, Capote et al. in a similar study of 154 patients found the incidence to be 0%, Keski-Säntti et al. observed an incidence of 34% [9] while for the early study of Bonnardot L et al. the incidence was up to 47% [10], close to the 45% found on Byers et al. study [11]. This corroborates the fact that the clinical examination and the CT scan are not enough for a reliable nodal staging [10] and put the light on the importance to look for constant predictor factors of these metastases.

Also, this observation impacts on the medical care as long as it can lead to an increase of the use of selective neck dissection for the N0 patients. Also, a protocol of postoperative radiotherapy can be proposed when positive nodes are found, eventually combined with concomitant chemotherapy if the patient presents another poor diagnosis factor [10].

If age is a risk factor of the tongue SCC, concerning predominantly the 60 years old and over, even if its incidence increased among young people, especially women, over the past 20 years, next to the toxic behaviours like alcohol excess and heavy smoking [3], these parameters don't seem to influence the occurrence of cervical nodal metastases on our study, as well as on J A Woolgar et al. study where there was no significant difference in relation to sex or age [12].

The main factors that emerged as predictive of the appearance of occult cervical nodal metastases and a worse prognosis in our study are the tumor depth invasion and the muscular invasion. Indeed, the depth invasion is introduced in the AJCC Cancer Staging Manual, 8th edition, defined as the distance from the deepest level of invasion to the reconstructed mucosal surface and classified as lesl invasive if ≤ 5 mm, moderate invasive if > 6 mm and <10 mm and deeply invasive if ≥ 10 mm [13]. A multicentric study demonstrated a better prognosis evaluation considering the optimal cut point as 5 mm in T1, 10 mm in T2, T3 and T4 [14]. Also, the extrinsic or deep muscular invasion may no longer be considered as a criterion of T4 as long as it is directly integrated in the depth invasion notion [15].

Also, the Tam et al. study confirmed that the depth of invasion of the tumor was an independent predictive factor of overall survival (OS) and disease-specific survival (DSS), demonstrating that the optimal cut-point, specifically for lateral tongue SCC, was 7,25 for OS and 8 mm for DSS, in contrast to the usual depth of 4 mm described by Kligerman et al. [16].

In parallel, the Jin Wu-Long et al. study also found a statistically significant correlation between the occurrence of occult nodal metastases and the depth invasion since the rate of these metastases was higher with the tumors invading the muscular layer, more than with the ones invading the submucosal layer, more than with these that do not cross the mucosal layer [7]. These observations were confirmed by the Chaudhary et al. study [9].

It is important to outline the difference between tumor thickness and depth of invasion. The indian study led by Arora et al. found that only the depth of invasion was a predictor of nodal metastases and a 4 mm cutoff point was revealed optimal. But, these results included all oral cavity subsites and the elective neck dissection was not performed for the totality of patients [17].

The study of Faisal et al. showed a significant increase of the risk of occult nodal metastasis (53%) with a decrease in 5 year survival to 45% secondary to the increase of tumor depth of invasion, particulary >10 mm. However, the patients with a tumor <5 mm of depth invasion had occult nodal metastases in 23% of the cases, confirming the importance of elective neck dissection even in early stages [13].

In addition to this first parameter, the incidence of occult nodal metastases seemed to be related to the increasing pathological grading of the tumor, as described on the Chaudhary et al. study [9], the M Russolo et al. study [5], and the Jin Wu-Long et al. study [7] where the metastases were linked to a higher pathological grade and a poorer degree of differentiation.

The perineural and vascular invasion was not analyzed on our work but, on the J A Woolgar et al. study, they had a significant independent value in predicting metastases, besides, these parameters are included in the histologic malignancy grading system they propose whose total score over 17 suggests a strong possibility of cervical metastases [12]. They are also a part of the Brandwein histologic risk model score which seems to have a significant predictive power on the recurrences and the overall survival rate for patients with oral squamous cell carcinomas [18]. Also, these two factors, vascular infiltration, more frequently identified than perineural invasion, have been recognized in multiple multivariate studies as a predictive factor of increasing the risk of lymph node and distant metastases and a major indication for oncologic adjuvant therapy [6].

Byers e*t al.* study proposed a model that can predict the probability of nodal involvement based on three factors that can explain the variability in pathologic node status. These factors are: depth of muscle invasion, N stage (N0 versus N1 versus N2 N3) and degree of tumor differentiation (well versus moderate versus poor). The perineural invasion (yes versus no) can also be cited even if omitted in the following study [11].

The tumor form appeared to play a role while the metastases used to occur more frequently with the infiltrative form, than with the ulcerative form, than with the exophytic form [7]. In clinical terms, for the same lesion on the surface, an ulcerative tumor will have a worse prognosis than an exophytic one [15], which can be explained by the relation between the tumor form and the depth invasion that was discussed earlier.

Also, the tumor site didn't seem to influence the nodal metastases [12]. Regarding the tumor size, the Jin Wu-Long et al. study, as well as ours, didn't find a significant correlation between the superficial size and the cervical metastases [7]. However, on the J A Woolgar et al. study the laboratory measurements of the tumor size showed significant differences between patients with and without metastases; but, the tumor thickness still more important [12].

Anatomically, according to Jin Wu-Long et al. study, the most common region with occult nodal metastases is the ipsilateral level II, followed by the level III, then by the levels I and IV. So, the ipsilateral levels I to III are the most important regions to be dissected, putting the supraomohyoid neck dissection as the first elective treatment [7].

An interesting strength of our present study is the clearly sub-sectioned description of the results with clinical data, while its limitations can be the small number of patients and the non-assessment of some contributory parameters such as the perineural invasion.

## 4 Conclusion

To sum up, it is clear that there still no consistent statistically factor that can surely predict the occult cervical nodal metastases of the tongue SCC. Advances in imaging technology, use of biomarkers, and the role of sentinel node biopsy require further research and validation.

For now, according of our study and the findings in literature, based on the study of these criteria, depth and muscular invasion, low differentiated, highly graded tumors should warn from the high incidence of occult cervical nodal metastases and should recommend an elective neck dissection in all cases of N0 tongue SCC [9].

## 6 Provenance and peer review

Not commissioned, externally peer-reviewed.

## Declaration of competing interest

None.

## Registration of research studies

Name of the registry: Research Registry.

Unique Identifying number or registration ID: 5887.

Hyperlink to your specific registration (must be publicly accessible and will be checked):

https://www.researchregistry.com/browse-the-registry#home/registrationdetails/5f307a2656df580015905dbb/
